# The Unusual Tribological Properties of Graphene/Antimonene Heterojunctions: A First-Principles Investigation

**DOI:** 10.3390/ma14051201

**Published:** 2021-03-04

**Authors:** Xian Jiang, Zhibin Lu, Renhui Zhang

**Affiliations:** 1 School of Materials Science and Engineering, East China Jiaotong University, Nanchang 330013, China; jiangxian@ecjtu.edu.cn; 2 College of Science, Lanzhou University of Technology, Lanzhou 730050, China; zblu@licp.cas.cn

**Keywords:** heterojunctions, first-principles, superlubricity, work of separation

## Abstract

The extremely low friction between incommensurate two-dimensional (2D) materials has drawn more attention in the recent years. Structural superlubricity is a fascinating tribological phenomenon that is achieved in 2D heterojunctions despite the aligned or misaligned contacts that occur due to the disappearance of the lateral interactions between two incommensurate contacting surfaces. In this study, using the first-principles method, we report the computational realization of structural superlubricity for graphene/antimonene heterojunctions at the nanoscale. The calculated results clearly demonstrate that structural superlubricity between graphene and antimonene monolayers could be achieved under the misaligned contacts. The structural superlubricity is mainly attributed to lower work of separation, which maintains superlow friction coefficients.

## 1. Introduction

Recently, two-dimensional (2D) materials, such as graphene and MS_2_ (M = W and Mo) have exhibited excellent tribological performance [1,2] due to their unique layered structure. Besides, antimonene, a new type of 2D material, has been successfully produced using the van der Waals epitaxy and liquid phase-based ultrasonic-assisted stripping method [3], and its light resistance was superior to graphene. 2D antimonene shows superior electrochemical properties [4,5]. However, the lubricity of 2D antimonene has so far been rarely reported. SnSe flat monolayers may exhibit low friction due to being easily sheared, as reported by Ashton et al. [6]. Similarily to that of SnSe, 2D antimonene was easily sheared due to its low exfoliation energy of 236 meV [7], therefore, 2D antimonene should achieve low friction.

The heterojunctions built from the different 2D materials would help with reducing the exfoliation energy and achieving superlubricity. Indeed, Mandelli and Leven et al. pointed out that graphene/h-BN heterojunctions would be a route to robust superlubricity [8,9]. They declared that the enhanced incommensurability effects would greatly reduce the friction coefficient in misaligned contacts. Besides, the robust microscale superlubricity in graphite/h-BN layered heterojunctions was found by Song et al. [10]. They demonstrated that structural superlubricity could be maintained, even when the aligned contact applied external loads. Thus, for 2D heterojunctions, the structural superlubricity would be achieved even for the aligned or misaligned contacts. Antimonene is a special 2D material, and if 2D heterojunctions were built with graphene, one may naturally wonder: could structural superlubricity be achieved?

Motivated by our previous work [11], we used the first-principles method to investigate the properties of graphene/antimonene heterojunctions, which focused on their structural superlubricity. We found that the work of separation (W_sep_) played an important role in obtaining superlow friction coefficient. Structural superlubricity could be achieved under the misaligned contacts. We discussed the unusually tribological behavior to explain what the most important factors for structural superlubricity between graphene and antimonene were.

## 2. Methods

**Figure 1:**
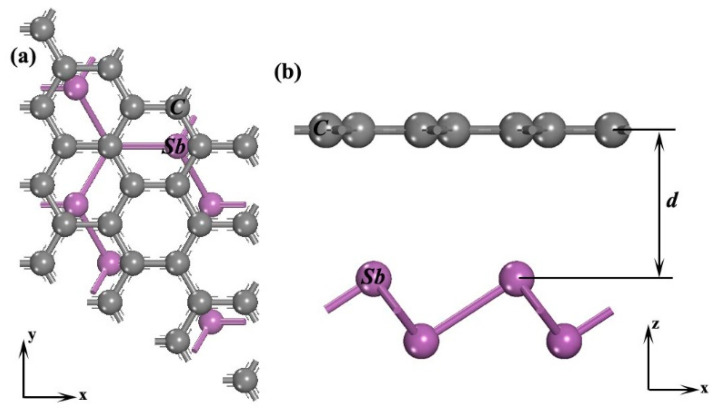
The calculated models: (**a**) top view and (**b**) side view. Note, d is interface distance along the *z* axis.

All calculations were carried out using the CASTEP code [12]. Based on Grimme’s method [13], the van der Waals interactions are well considered under generalized gradient approximation (GGA) with the PBE-D scheme [14]. In the Ortmann, Bechstedt, and Schmidt (OBS) scheme, R_vdW_ is set as 0.77 (alpha = 1.76 Å^3^) and 1.38 Å (alpha = 6.6 Å^3^) for carbon and antimony, *n* = 8, and a damping constant of λ = 7.5 × 10^−4^ is chosen in this work [15]. During geometry optimization, the cutoff energy of 420 eV is selected for the plane-wave basis set. For k-point sampling, 25 × 25 × 1 Monkhorst-Pack mesh is applied. The models are relaxed until the total energy tolerances and force are less than 2.0 × 10^−5^ eV/atom and 0.1 eV/Å. According to the match methods in Refs. [16,17], there is only one possible Moiré pattern graphene (0 0 −1) surface with a 3 × 3 supercell (7.38 Å × 7.38 Å) and antimonene (0 0 −1) surface with a 1 × 1 unit cell (7.3 Å × 7.3 Å), as displayed in Figure 1, with in-plane hexagonal edges aligned in the same orientation and with less than 1% lattice mismatch. Periodic boundary conditions are added in the x-y plane, the moving direction is only applied along the x direction for calculating the work of separation due to the same potential energy between moving along the *x* and *y* axis. To avoid the interactions between periodic replicas, the vacuum slabs of 20 Å are added along the *z* axis. The potential energy surfaces (PESs) are sketched by collecting the interlayer interaction energy of the upper graphene monolayer translation over the lower antimonene monolayer for the variable relative lateral positions [18]. The charge is calculated based on the Mulliken population analysis [19].

In our previous work, adhesion between two contact surfaces could be well characterized using the work of separation (*W_sep_*) [20]. Thus, the *W_sep_* is calculated as follows:

(1) $$W_{sep}=\left(E_{Anti}^{tot}+E_{Gr}^{tot}-E_{Gr-Anti}^{tot}\right)/A,$$

where $E_{Anti}^{tot}$ and $E_{Gr}^{tot}$ is the total energy of antimonene and graphene, $E_{Gr-Anti}^{tot}$ is the total energy of graphene/antimonene heterojunctions, and *A* is the interfacial area. The maximum shear force could be calculated by the Equation (2):

(2) $$f_{x,y}{|}_{max}=-\frac{\partial W_{sep}}{\partial x,y}.$$

Subsequently, we compress the graphene/antimonene heterojunctions in order to investigate the effect of the normal load on the tribological properties. $F_{z}=-\frac{\partial V\left(z\right)}{\partial z}$ replaces the force along *z* axis, where *V(z)* represents the potential energy with variable interface distances along *z* axis.

## 3. Results

### 3.1. Potential Energy and Force with Variable Interface Distance along the z Axis

**Figure 2:**
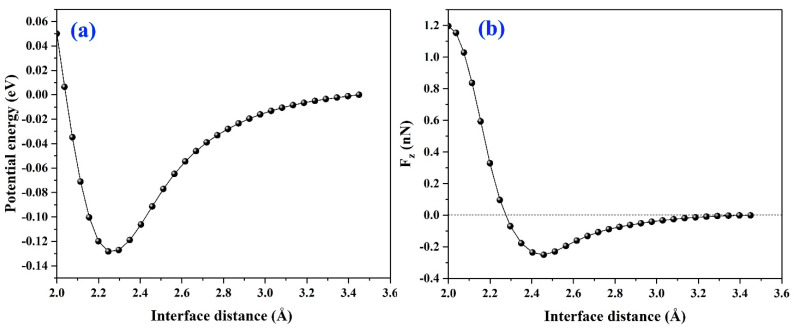
(**a**) Potential energy and (**b**) force as a function of the interface distance d along *z* axis.

The potential energy (PE) curve, with respect to the interface distance d, is plotted in Figure 2a. One minimum point is observed at *d* = 2.25 Å, and as *d* < 2.25 Å, where the potential energy sharply increases. The derivative of potential energy (F_z_) displayed in Figure 2b shows that as 2.25 Å < *d* < 3.26 Å, F_z_ corresponds to the attractive force, rather than corresponding to the repulsive force as *d* < 2.25 Å.

**Figure 3:**
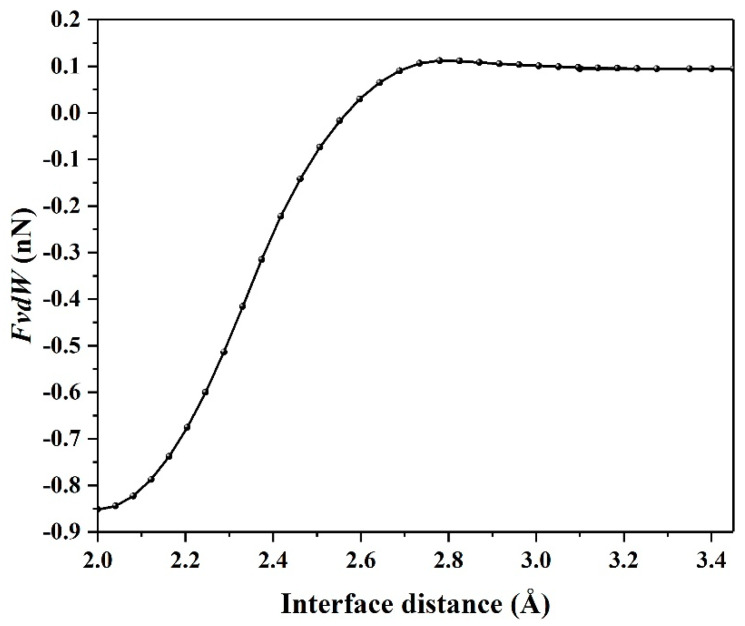
The van der Waals force *F_vdW_* with respect to interface distance d.

Next, the van der Waals force (*F_vdW_*) is calculated, as shown in Figure 3, the forces for graphene/antimonene heterojunctions are listed in Table 1. Combining Table 1 with Figure 2b and Figure 3, we find that they obey the regulation as follows: $F_{z}\vec{i}=F_{N}\vec{j}+F_{vdW}\vec{k}$, where *F_N_* represents the pressure along *z* axis. $\vec{i},\;\vec{j},\;\vec{k\;}$ replaces the direction vectors. Note that the interlayer distance is kept fixed at 3.45, 2.25, and 2.0 Å to obtain the force values of *F_z_*, *F_vdW_*, and *F_N_*.

It clearly indicates that the van der Waals force plays an important role in balancing the graphene/antimonene heterojunctions by increasing the interface distance. The negative van der Waals force exhibits a repulsion force at *d* < 2.55 Å, but the van der Waals force also shows positive values at 2.55 Å < *d* < 3.45 Å, indicating an attractive force between graphene and antimonene.

### 3.2. Work of Separation with Respect to Interface Distance along z Axis and Three-Dimensional Work of Separation with Respect to x and y

**Figure 4:**
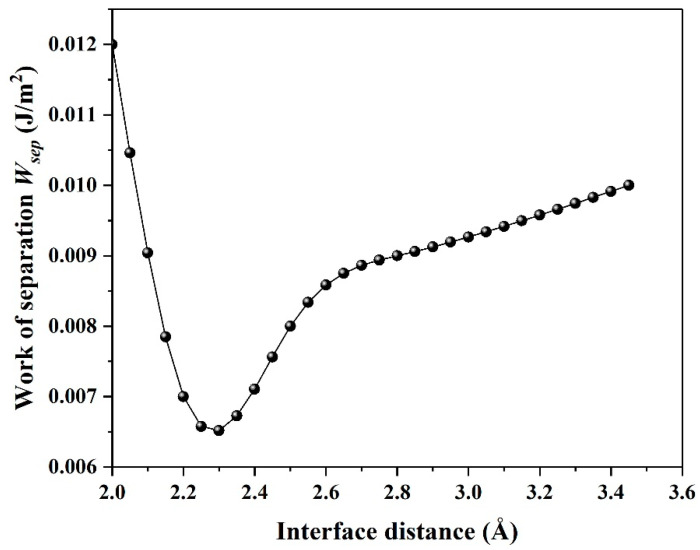
Work of separation (*W_sep_*) along the *z* axis with respect to interface distance d.

In general, adhesion between two solid surfaces is typically characterized by the work of separation *W_sep_*, as shown in Figure 4. Generally, the value of *W_sep_* could well reflect the adhesive strength between two contact surfaces. Here, *W_sep_* shows small values with decreasing the interface distance, indicating lower adhesive strength between graphene and antimonene.

**Figure 5:**
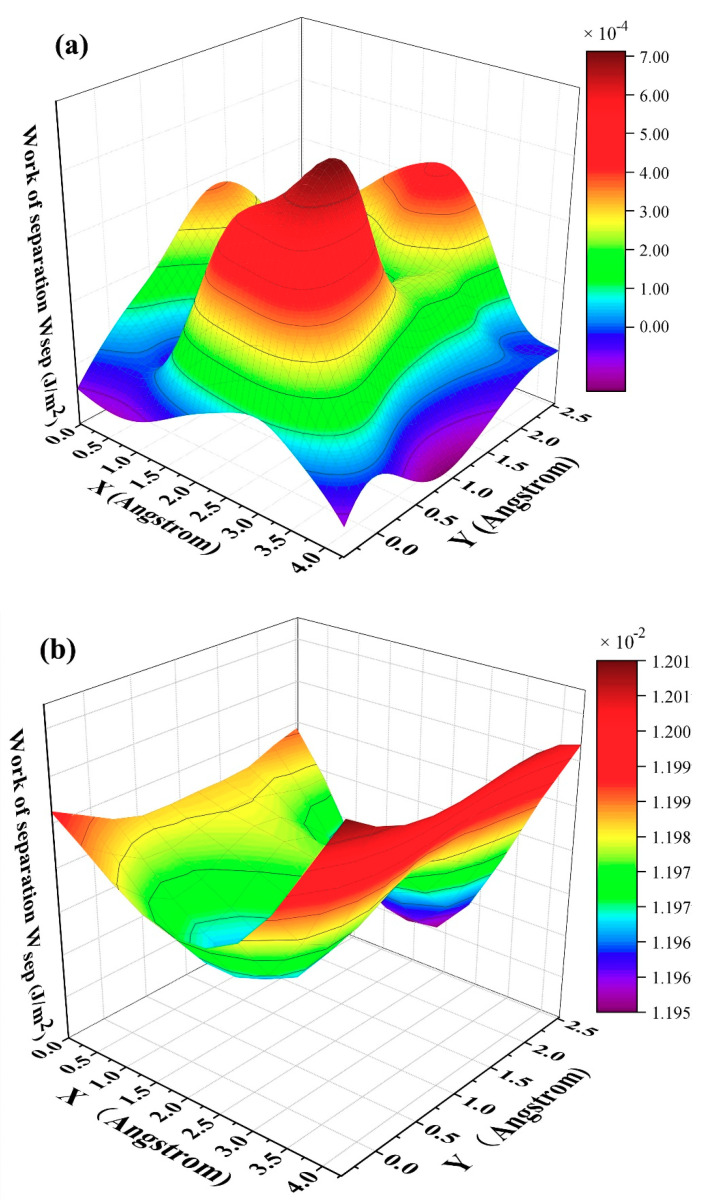

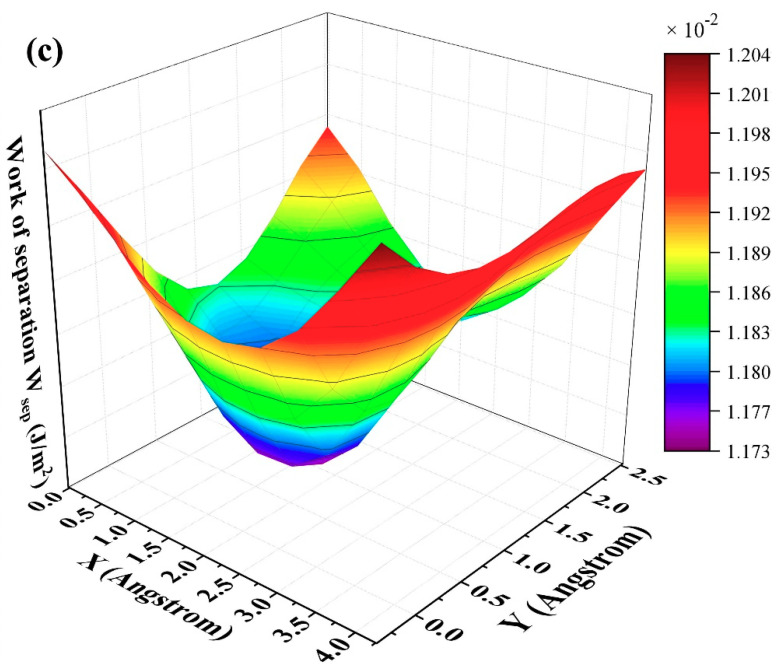
Three-dimensional work of separation with respect to interface distance d: (**a**) 3.45 Å, (**b**) 2.25 Å, and (**c**) 2.0 Å moving along the *x* and *y* axis. Note, the *z* axis represents the work of separation, and the *x* and *y* axis represent the moving direction.

Because work of separation (*W_sep_*) is an important physical factor for investigating the tribological properties, we thus calculate three-dimensional (3D) *W_sep_*, as plotted in Figure 5. Qi and Cui et al. reported that the minimum *W_sep_* corresponds to a low friction coefficient under the static state [21,22]. In our previous work, we confirmed that the theory obtained by Qi and Cui et al. is also applied for dynamic state [20]. As shown in Figure 5a–c, *W_sep_* has no concern with interface distance and exhibits small values less than 0.012 J/m^2^. In order to further understand this phenomenon, the electron density and density of states are calculated subsequently.

### 3.3. Electron Density Difference and Density of States Analysis

**Figure 6:**
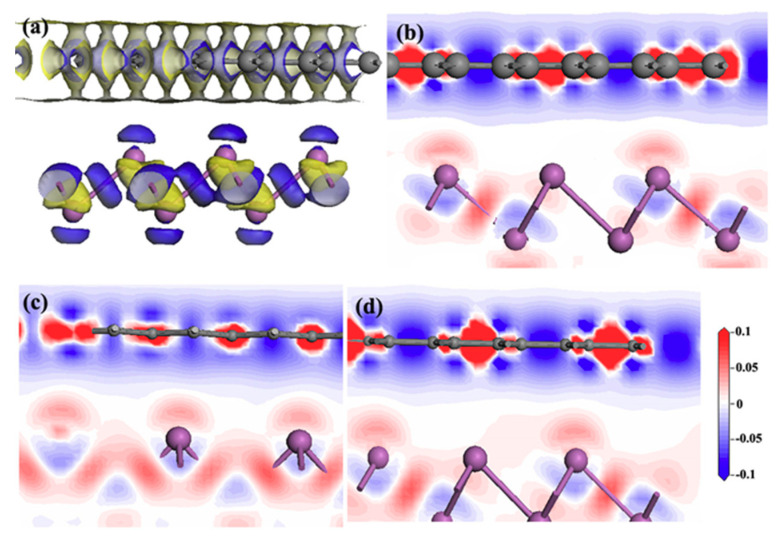
(**a**) Three-dimensional electron density difference of graphene/antimonene heterojunctions. There is a two-dimensional electron density difference of graphene/antimonene heterojunctions with respect to the interface distance: (**b**) 3.45 Å, (**c**) 2.25 Å, and (**d**) 2.0 Å.

Electron density difference Δ*ρ* has often been used to analyze electronic transitions between two interfaces. The depletion and accumulation of electron density at the atomic interface would closely affect the tribological performance of the tribosystems. Thus, in order to further investigate the charge distribution of graphene/antimonene heterojunctions, the 3D electron density difference of graphene/antimonene heterojunctions is calculated, as shown in Figure 6. In Figure 6a, the blue and orange color represents the positive and negative isosurfaces. Figure 6b–d shows the 2D electron density difference. We find that around the interface, Sb atoms lose electrons and bond with its neighboring ones, and there are many electrons near the interface for the graphene slab. Thus, there exists an electronic enrichment area between the graphene and antimonene interface.

**Figure 7:**
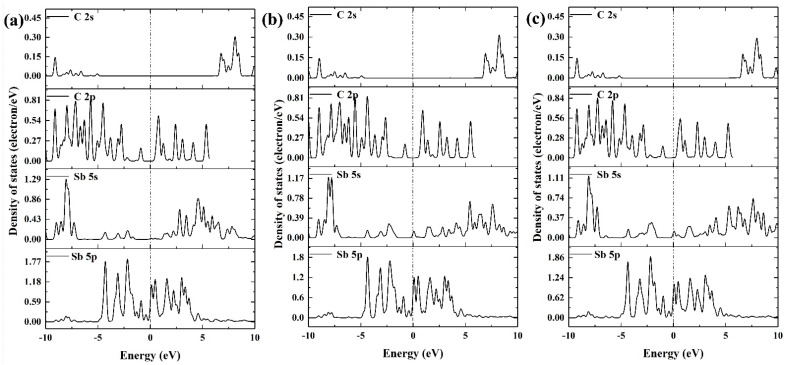
The density of states with respect to the interface distance d for graphene/antimonene heterojunctions: (**a**) 2.0 Å, (**b**) 2.25 Å, and (**c**) 3.45 Å.

**Figure 8:**
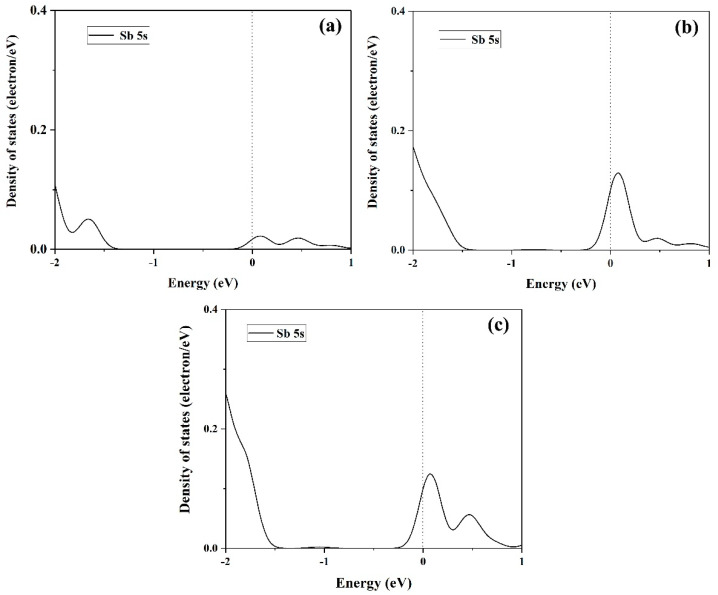
The density of states of Sb 5s ranging from −2 to 1 eV for graphene/antimonene heterojunctions: (**a**) 2.0 Å, (**b**) 2.25 Å, and (**c**) 3.45 Å.

Figure 7 shows the density of states for different interface distance. The decrease of interface distance has no effect on the density of the states of C 2s and C 2p. However, for valence bands of Sb 5s states, the split states are found to decrease the interface distance, and the state at about 0 eV is negatively shifted to about −1.6 eV, indicating that a strong repulsive interaction exists between graphene and antimonene, which is consistent with the analysis of electron density difference. Figure 8 shows the density of states (DOS) of Sb 5s ranging from −2 to 1 eV. For Figure 8a–c, in the valence band, the DOS gradually moves downward and is enhanced around −2 eV.

### 3.4. Potential Energy Surfaces Analysis

**Figure 9:**
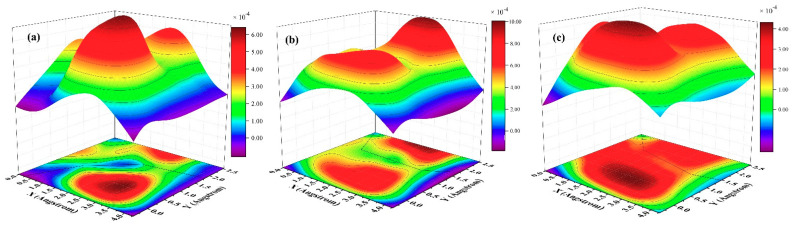
Three-dimensional potential energy surfaces with respect to interface distance d for graphene/antimonene heterojunctions: (**a**) 3.45 Å, (**b**) 2.25 Å, and (**c**) 2.0 Å. Note, the color scale refers to the energy range of PES corrugation (in eV/cell) and is plotted on the right.

**Figure 10:**
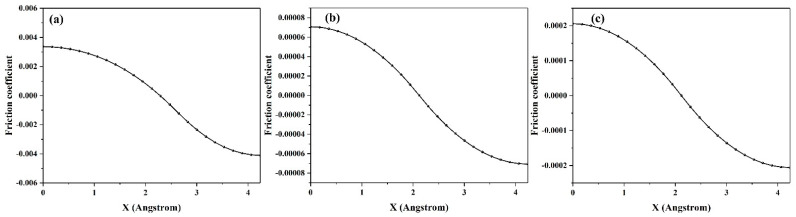
The friction coefficient along the *x* axis for graphene/antimonene heterojunctions at variable interface distance d: (**a**) 3.45 Å, (**b**) 2.25 Å, and (**c**) 2.0 Å.

Subsequently, 3D potential energy surfaces (PESs) are calculated with respect to interface distance d, as shown in Figure 9. From Figure 9a–c, only one low friction path is observed. The shape of PESs changes by decreasing the interface distance. Low friction paths are observed sliding along the valleys. The values of potential energies are less than 0.001 eV/cell. Combining Figure 5, Figure 6 and Figure 9, we can confirm that the structural superlubricity could be achieved for graphene/antimonene heterojunctions. In our previous work, the maximin shear force ($f_{x,y}{|}_{max}$) could correspond to the extremely static friction force [20]. The friction coefficient could be calculated by: $\mu_{x,y}=\;\frac{f_{x,y}{|}_{max}}{F_{N}}$. Combining Equations (1) and (2), μx,y=−∂Wsep∂x,yFN. Because the low friction paths mainly distribute along the *x* axis, we only calculate the friction coefficient along the *x* axis, as shown in Figure 10. In Figure 10a–c, the systems exhibit a superlow friction coefficient (0.00007−0.0002) moving along the *x* axis. It implies that structural superlubricity is achieved for the graphene/antimonene heterojunctions.

## 4. Discussion

In this work, we successfully investigated the tribological properties of graphene/antimonene heterojunctions. We found that structural superlubricity can be achieved between a graphene and antimonene interface. We mainly proceed from three aspects to answer this interesting phenomenon. As shown in Figure 4 and Figure 5, the system exhibits lower *W_sep_* (about 0.01 J/m^2^), and the calculated values are less than that in previous work (0.02–0.2 J/m^2^) [21,22,23,24]. The figures also point out that low *W_sep_* corresponds to a low friction coefficient. Thus, we can conclude that lower *W_sep_* should be one important factor for structural superlubricity. On the other hand, the electronic structure and density of states, as shown in Figure 6, Figure 7 and Figure 8, show strong repulsive interactions between graphene and antimonene. The strong repulsive interactions should provide a strong repulsive force to obtain superlow friction coefficient, which could be summarized as the other factor. Last, under the misaligned contacts, potential energy possesses the lowest values, as shown in Figure 9. That is, the system could easily slide over the low friction paths due to the lowest energy barriers. Therefore, we could confirm that the lowest energy barriers should be another factor for structural superlubricity. We expect that this investigation should give a positive guidance for the experimental investigations.

## 5. Conclusions

In summary, we have successfully probed the unusually tribological behavior of graphene/antimonene heterojunctions using first-principles calculations. We obtained the followed conclusions: (1) As *d* < 2.25 Å, where the potential energy sharply increases and F_z_ transfers from attractive force to repulsive one. (2) A lower work of separation is found for graphene/antimonene heterojunctions, which play an important role in obtaining structural superlubricity. (3) The strong repulsive interactions and lowest energy barriers could be summarized as two other factors for structural superlubricity. The calculated results exhibit that graphene/antimonene heterojunctions could be an appealing lubricant material. We believe that this work could provide positive guidance for further experimental investigations.

## Figures and Tables

**Table 1:** The forces (*F_z_*, *F_vdW_*, and *F_N_*) for graphene/antimonene heterojunctions with respect to interface distance d.

*z* (Å)	*F_z_* (nN)	*F_vdW_* (nN)	*F_N_* (nN)
3.45	0	0.09	0.09
2.25	0	0.6	0.6
2.0	1.88	0.85	1.03
